# Association between *MTHFR* gene polymorphisms and H-type hypertension in patients with ischemic stroke

**DOI:** 10.7717/peerj.20210

**Published:** 2025-10-10

**Authors:** Bo Zhou, Tingting Yang, Shicang An, Qike Xu, Yuna Liang, Xiangyang An

**Affiliations:** 1 Postdoctoral Workstation, The Affiliated Taian City Central Hospital of Qingdao University, Taian, Shandong, China; 2 General Obstetrics and Gynecology, The Affiliated Taian City Central Hospital of Qingdao University, Taian, Shandong, China; 3 Laboratory Medicine Department, The Affiliated Taian City Central Hospital of Qingdao University, Taian, Shandong, China; 4 Radiology Department, The Affiliated Taian City Central Hospital of Qingdao University, Taian, Shandong, China; 5 Ultrasound Diagnosis and Treatment Department, The Affiliated Taian City Central Hospital of Qingdao University, Taian, Shandong, China

**Keywords:** MTHFR, Hypertension, Homocysteine, Ischemic stroke

## Abstract

**Background:**

Methylenetetrahydrofolate reductase (*MTHFR*) is a key enzyme in homocysteine metabolism. Its 677C>T and 1298A>C polymorphisms can reduce enzyme activity, potentially elevating homocysteine levels. H-type hypertension (hypertension with homocysteine ≥10 μmol/L) is an important risk factor for ischemic stroke, and its synergistic effect exacerbates vascular damage. However, the association between these *MTHFR* polymorphisms and elevated homocysteine levels in patients with hypertension complicated by ischemic stroke remains unclear. This study aimed to investigate the association between *MTHFR* gene polymorphisms and H-type hypertension in patients with ischemic stroke.

**Methods:**

A total of 215 patients with ischemic stroke and hypertension admitted to the Department of Neurology at the Taian City Central Hospital from June 2021 to December 2022 were enrolled. General clinical data and biochemical indicators were collected. *MTHFR* genotyping was performed using a universal sequencing kit and a TL998A fluorescence detector. Linkage disequilibrium was analyzed *via* SHEsis software. Statistical analyses were conducted using SPSS 25.0. *P* < 0.05 indicates that the difference is statistically significant.

**Results:**

Among patients with ischemic stroke combined with hypertension in this region, the proportion of H-type hypertension was 89.3%. The proportion of males in the H-type hypertension group was significantly higher than in the non-H-type hypertension group (*P* < 0.05). The genotype and allele distributions of *MTHFR* *(677C>T) (risk* allele: T) differed significantly between groups (*P* < 0.05): the H-type group had a higher frequency of the TT genotype (47.4% *vs*. 17.4%) and T allele (67.2% *vs*. 50.0%). Multivariate logistic regression analysis showed that the *MTHFR* *(677C>T)* TT genotype was an independent risk factor for H-type hypertension (*P* = 0.021, *OR* = 2.615, 95%CI [1.154–5.926]*)*. For haplotypes with a frequency >3%, there were three haplotypes of *MTHFR* *(677C>T)/(1298A>C)*. The C-A haplotype was a protective factor for H-type hypertension (*P* = 0. 028, *OR* = 0.485, 95%CI [0.252–0.934]), while the T-A haplotype was a risk factor (*P* = 0.022, *OR* = 2.029, 95%CI [1.096–3.756]).

**Conclusion:**

In patients with ischemic stroke, the *MTHFR* *(677C>T)* TT genotype is an independent risk factor for H-type hypertension. For haplotypes with a frequency >3%, the C-A haplotype was a protective factor for H-type hypertension, whereas the T-A haplotype was a risk factor.

## Introduction

With societal development, lifestyle changes, accelerated population aging, and the increasingly prominent unhealthy lifestyles, cerebrovascular disease has become one of the main causes of death (Zhou et al., 2019). Hypertension is the most common comorbidity in hospitalized stroke patients, accounting for approximately 66.6% (National Center for Cardiovascular Diseases, China Cardiovascular Health and Disease Report Writing Group, 2024). The number of hypertensive patients in China has reached 245 million (National Center for Cardiovascular Diseases, China Cardiovascular Health and Disease Report Writing Group, 2024), with H-type hypertension accounting for about 75% (Chen et al., 2023; Qian et al., 2021). H-type hypertension is defined as essential hypertension with a homocysteine concentration ≥10 μmol/L. The risk of cardiovascular and cerebrovascular diseases in H-type hypertension patients is five times higher than that in hypertension patients (two instances of resting systolic blood pressure ≥140 mmHg and/or diastolic blood pressure ≥90 mmHg or history of hypertension) (Zhang et al., 2023a). Studies have shown that H-type hypertension can significantly increase the incidence of ischemic stroke (Yang et al., 2023; Yu et al., 2023), and lead to higher mortality (Zhao et al., 2022). As an important risk factor for ischemic stroke, H-type hypertension and ischemic stroke exacerbate vascular damage through synergistic effects, significantly increasing the risk of stroke occurrence (Chen et al., 2025). In patients with H-type hypertension, elevated homocysteine levels increase stroke risk and are associated with the methylene tetrahydrofolate reductase (*MTHFR*) *C677T* polymorphism. The CC/CT genotype shows an increased risk (*HR* = 3.1) (Zhao et al., 2017). Therefore, exploring the factors influencing H-type hypertension is crucial for reducing cardiovascular and cerebrovascular events.

*MTHFR*, located on chromosome 1p63.6, is an important enzyme in folate and homocysteine metabolism (Raghubeer & Matsha, 2021). The *MTHFR (677C>T)* and *MTHFR (1298A>C)* are the common mutation sites in *MTHFR*, and exhibit a synergistic effect (Zhou et al., 2025). Concurrent mutations may further reduce enzyme activity. The *MTHFR (677C>T)* is a mutation at nucleotide 677 from C to T, which leads to a change in the encoded protein to change from alanine to valine. The mutant heterozygous enzyme activity decreased by about 35%, while the mutant homozygous enzyme activity decreased by about 70% (Rai & Kumar, 2021). *MTHFR (1298A>C)* is a mutation at nucleotide 1298 from A to C, which encodes a protein changed from glutamate to alanine. The mutant heterozygous enzyme activity decreased by about 10%, while the mutant homozygous enzyme activity decreased by about 40% (Li et al., 2022). There is a synergistic effect between the two mutations of the same enzyme, and the simultaneous mutation could further decrease the enzyme activity (Ginani et al., 2023).

Currently, most studies mainly focus on the association between the *MTHFR (677C>T)* polymorphism and H-type hypertension. The *MTHFR (677C>T)* polymorphism is significantly associated with an increased risk of H-type hypertension except in the overdominant model (Kong et al., 2022). However, few studies have explored the association between the *MTHFR (677C>T)* and/or *MTHFR (1298A>C)* and H-type hypertension. This study aims to investigate the association between the *MTHFR (677C>T)* and/or *MTHFR (1298A>C)* genotypes and H-type hypertension.

## Materials and Methods

### Participants

A total of 215 ischemic stroke patients with hypertension who were hospitalized in the Neuro-Brain Center of Taian City Central Hospital from June 2021 to December 2022 were enrolled. Patients were divided into two groups based on homocysteine levels: the H-type hypertension group (*n* = 192) with an average age of 62.6 ± 11.2 years (135 males, 57 females), and the non-H-type hypertension group (*n* = 23) with a mean age of 58.4 ± 11.5 years (11 males, 12 females).

Inclusion criteria: (1) Patients meeting the diagnostic criteria for hypertension in the Revised Chinese Hypertension Management Guidelines (2024 revision) (Chinese Hypertension Prevention and Treatment Guidelines Revision Committee et al., 2024), (2) Patients who underwent homocysteine testing during hospitalization, (3) Patients who underwent *MTHFR* gene testing during hospitalization. Exclusion Criteria: (1) Patients with thyroid disease, (2) Patients with blood system diseases (anemia, leukemia, platelet abnormalities, hemorrhagic diseases), (3) Incomplete data, (4) Patients with hepatic and renal insufficiency.

The study was approved by the Ethics Committee of the Affiliated Taian City Central Hospital of Qingdao University (No. 2021-06-50, Date: 11.05.2021). Written informed consent was obtained from the patients or their family members.

### Reagents and instruments

Reagents and instruments for study: Universal sequencing reaction kits, nucleic acid purification reagents, NH_4_Cl, sterilized water for injection (500 mL), TL998A fluorescence detector, Eppendorf high-speed centrifuge 5,418, Eppendorf pipettes (10, 200, 1,000 μL), centrifuge (1.5 mL), pipette tips (10, 200, 1,000 μL), EDTA anticoagulation centrifuge tubes (2 mL).

### Data collection

The general clinical data of the subjects were collected, including age, gender, diabetes, smoking, and drinking. Biochemical indicators were collected, including total cholesterol (TC), triglyceride (TG), low-density lipoprotein cholesterol (LDL-C), high-density lipoprotein cholesterol (HDL-C), fasting blood glucose (FBG), uric acid (UA), apolipoprotein A1 (ApoA1), apolipoprotein B (ApoB), and homocysteine concentrations (Hcy).

### Specimens collection

On the day of admission or the morning of the second day, collect 1.5 mL of fasting peripheral venous blood in an EDTA anticoagulant tube, mix thoroughly, and store at 4 °C to prevent hemolysis or coagulation. The maximum storage time is 24 h.

### Detecting the *MTHFR* gene polymorphism

(1) Add 1 mL of ammonium chloride to the centrifuge tube, then add 150 μL of venous blood and let stand for 5 min, (2) centrifuge at 3,000 rpm for 5 min, and discard the supernatant, (3) add 50 μL of nucleic acid purification reagent and mix, (4) add 1.5 μL of suspension to the corresponding universal kit for sequencing reaction. Check for any liquid residue at the front of the pipette tip and firmly fix the lid. Invert the tube several times to ensure thorough mixing, and gently tap the tube wall to remove any bubbles on the liquid surface. Use a micro centrifuge to briefly remove droplets attached to the tube wall, and test the resulting mixture using a machine according to the software number, (5) use the TL998A fluorescence detector (Xi’an Tianlong Science and Technology Co., Ltd.) for testing, (6) check the fluorescence spectrum image for gene typing.

### Statistical analysis

The Hardy-Weinberg equilibrium test was used to verify whether the sample was representative of the general population. Linkage disequilibrium (LD) was analyzed using the online tool SHEsis (Shi & He, 2005). All statistical analyses were performed using SPSS 25.0. Continuous data conforming to a normal distribution were presented as mean ± standard deviation ( ${\bar {\rm x}}$ ± SD); continuous data conforming to a normal distribution were presented as M (*P*_25_, *P*_75_). Categorized data were represented as counts (%). Comparison between groups was performed using the chi-square test, t-test, and rank-sum test. Multivariate logistic regression analysis was used to analyze the association between *MTHFR (677C>T)* and/or *MTHFR (1298A>C)* and H-type hypertension. *P* < 0.05 indicates that the difference was statistically significant.

## Results

### Hardy-Weinberg genetic equilibrium

The Hardy-Weinberg genetic equilibrium results show that the distribution of *MTHFR (677C>T)* and *MTHFR (1298A>C)* genotype in two groups are in genetic balance (non-H-type hypertension group, *χ*^*2*^= 1.415, *P* = 0.493 and *χ*^*2*^= 0.107, *P* = 0.743. H-type hypertension group, *χ*^*2*^= 0.838, *P* = 0.658 and *χ*^*2*^= 1.47, *P* = 0.48). These results indicate that the included subjects are representative of the general population.

### Characteristics of the study population

Among the 215 patients enrolled in this study, 192 patients had H-type hypertension, accounting for 89.3% of the hypertensive population. There were no statistically significant differences between the two groups in terms of age, diabetes, smoking, drinking, TG, TC, LDL-C, HDL-C, FPG, UA, ApoA1, and ApoB between the two groups (*P* > 0.05). However, the proportion of males in the H-type hypertension group was significantly higher than that in the non-H-type hypertension group (*P* < 0.05) (Table 1).

**Table 1:** Baseline characteristics.

Characteristics	H-type hypertension group (192)	Non-H-type hypertension group (23)	χ^*2*^*/t/z*	*P*
Age (years)	62.6 ± 11.2	58.4 ± 11.5	1.696	0.091
gender (*n*, %)	135 (70.3%)	11 (47.8%)	4.765	0.029
Diabetes (*n*, %)	49 (25.5%)	5 (21.7%)	0.156	0.693
Smoking (*n*, %)	67 (34.9%)	6 (26.1%)	0.711	0.399
Drinking (*n*, %)	72 (37.5%)	8 (34.8%)	0.065	0.799
TG (mmol/L)	4.46 ± 1.04	4.41 ± 1.14	0.246	0.806
TC (mmol/L)	1.23 (0.89, 1.75)	1.56 ± 0.64	1.282	0.2
LDL-C (mmol/L)	2.83 ± 0.84	2.83 ± 0.94	0.06	0.995
HDL-C (mmol/L)	1.21 ± 0.28	1.18 ± 0.33	0.485	0.628
FBG (mmol/L)	5.72 (4.94, 7.07)	5.48 (4.81, 6.34)	0.922	0.356
UA (mmol/L)	285 (236, 336)	267 ± 79.5	0.782	0.434
ApoA1 (g/L)	1.22 ± 0.17	1.23 ± 0.19	0.245	0.807
ApoB (g/L)	0.96 ± 0.23	0.97 ± 0.27	0.171	0.864

**Note:**

TG, Triglycerides; TC, Total cholesterol; LDL-C, Low-density lipoprotein cholesterol; HDL-C, High-density lipoprotein cholesterol; FPG, Fasting plasma glucose; UA, Uric acid; ApoA1, Apolipoprotein A1 and ApoB, Apolipoprotein B.

### Distribution of *MTHFR (677C>T)* and *MTHFR (1298A>C)* genotypes and alleles

The distribution frequency of *MTHFR (677C>T)* CC genotype and CT genotype in the non-H-type hypertension group was significantly higher than that in the H-type hypertension group, while the distribution frequency of the TT genotype was lower than that in the H-type hypertension group, and the difference was statistically significant (*P* < 0.05). The distribution frequency of the *MTHFR* C allele in the non-H-type hypertension group was significantly higher than that in the H-type hypertension group, and the difference was statistically significant (*P* < 0.05). However, there were no significant differences in the genotype or allele distributions of *MTHFR (1298A>C)* between the two groups (Table 2).

**Table 2:** The distribution of MTHFR (677C>T) and MTHFR (1298A>C) genotypes and alleles.

Gene	Genetype/ allele	*N* (%)	H-type hypertension group (%)	Non-H-type hypertension group (%)	*χ* ^*2*^	*P*
*MTHFR* *(677C>T)*						
	CC	29 (13.5%)	25 (13.0%)	4 (17.4%)	7.664	0.022
	CT	91 (42.3%)	76 (39.6%)	15 (65.2%)		
	TT	95 (44.2%)	91 (47.4%)	4 (17.4%)		
	C	149 (34.7%)	126 (32.8%)	23 (50.0%)	5.359	0.021
	T	281 (65.3%)	258 (67.2%)	23 (50.0%)		
*MTHFR* *(1298A>C)*						
	AA	164 (76.3%)	148 (77.1%)	16 (69.6%)	1.983	0.371
	AC	45 (20.9%)	38 (19.8%)	7 (30.4%)		
	CC	6 (2.8%)	6 (3.1%)	0 (0%)		
	A	373 (86.7%)	334 (87.0%)	39 (84.8%)	0.172	0.678
	C	57 (13.3%)	50 (13.0%)	7 (15.2%)		

### Logistic regression analysis of factors of H-type hypertension

Using H-type hypertension status as a dependent variable (non-H-type hypertension = 0, H-type hypertension = 1), a logistic regression analysis was conducted with gender (female = 0, male = 1), age, *MTHFR (677C>T)* genotype (CC = 0, CT = 1, TT = 2), *MTHFR (1298A>C)* genotype (AA = 0, AC = 1, CC = 2), diabetes (no = 0, yes = 1), smoking (no = 0, yes = 1), drinking (no = 0, yes = 1), TG, TC, LDL-C, HDL-C, FBG, UA, ApoA1 and ApoB as independent variables. The results showed that gender, age, and *MTHFR (677C>T)* TT genotype were significantly associated with H-type hypertension (*P* < 0.05). Gender (*P* = 0.018, *OR* = 4.845, 95%CI [1.309–17.939]) and age (*P* = 0.037, *OR* = 1.05, 95%CI [1.003–1.100]) were identified as independent risk factors. The *MTHFR (677C>T)* TT genotype was an independent risk factor for H-type hypertension (*P* = 0.021, *OR* = 2.615, 95%CI [1.154–5.926]) (Table 3).

**Table 3:** Multivariate logistic regression analysis of the influencing factors of H-type hypertension.

Variables	Group	β	SE	Wald	*P*	*OR*	*OR* (95%CI)
Gender	Female*						
		1.578	0.668	5.583	0.018	4.845	[1.309–17.939]
Age		0.049	0.024	4.353	0.037	1.05	[1.003–1.100]
*MTHFR (677C>T)*		0.961	0.417	5.302	0.021	2.615	[1.154–5.926]
*MTHFR (1298A>C)*		0.307	0.529	0.338	0.561	1.36	[0.482–3.832]
Diabetes	None*						
		0.072	0.636	0.013	0.91	1.075	[0.309–3.737]
Smoking	None*						
		−0.124	0.695	0.032	0.858	0.883	[0.226–3.450]
Drinking	None*						
		−0.409	0.647	0.399	0.527	0.664	[0.187–2.362]
TC		1.985	1.68	1.396	0.237	7.277	[0.270–195.915]
TG		−0.363	0.519	0.488	0.485	0.696	[0.251–1.925]
LDL-C		−0.981	1.527	0.412	0.521	0.375	[0.019–7.477]
FBG		0.013	0.109	0.014	0.906	1.013	[0.818–1.255]
UA		0.004	0.004	1.085	0.298	1.004	[0.997–1.011]
apoA1		−4.96	3.149	2.482	0.115	0.007	[0.000–3.357]
apoB		−3.839	3.967	0.936	0.333	0.022	[0.000–51.280]
HDL-C		0.397	3.094	0.016	0.898	1.487	[0.003–639.855]

**Note:**

* Control group

### Distribution of *MTHFR (677C>T)* and *MTHFR (1298A>C)* linked genotypes

In the H-type hypertension group, there are 7 linked genotypes of *MTHFR (677C>T)* and *MTHFR (1298A>C)*, which are CC/AA (5.2%), CC/AC (4.7%), CC/CC (3.1%), CT/AA (25.5%), CT/AC (21.7%), TT/AA (46.4%), and TT/AC (1.0%). The most frequent genotype was TT/AA (46.4%) (Table 4). In the non-H-type hypertension group, there are five linked genotypes of *MTHFR (677C>T)* and *MTHFR (1298A>C)*, which are CC/AA (8.7%), CC/AC (8.7%), CT/AA (43.5%), CT/AC (21.7%), TT/AA (17.4%). The highest frequency was CT/AA (43.5%) (Table 5).

**Table 4:** The distribution of MTHFR (677C>T) and MTHFR (1298A>C) linked genotypes in H-type hypertension group.

*MTHFR (677C>T)*		*MTHFR (1298A>C)*	
	AA	AC	CC
CC	10 (5.2%)	9 (4.7%)	6 (3.1%)
CT	49 (25.5%)	27 (21.7%)	0 (0%)
TT	89 (46.4%)	2 (1.0%)	0 (0%)

**Table 5:** The distribution of MTHFR (677C>T) and MTHFR (1298A>C) linked genotypes in non-H-type hypertension group.

*MTHFR (677C>T)*		*MTHFR (1298A>C)*	
	AA	AC	CC
CC	2 (8.7%)	2 (8.7%)	0 (0%)
CT	10 (43.5%)	5 (21.7%)	0 (0%)
TT	4 (17.4%)	0 (0%)	0 (0%)

### Relationship between *MTHFR (677C>T)* and *MTHFR (1298A>C)* interaction and H-type hypertension

**Figure 1:**
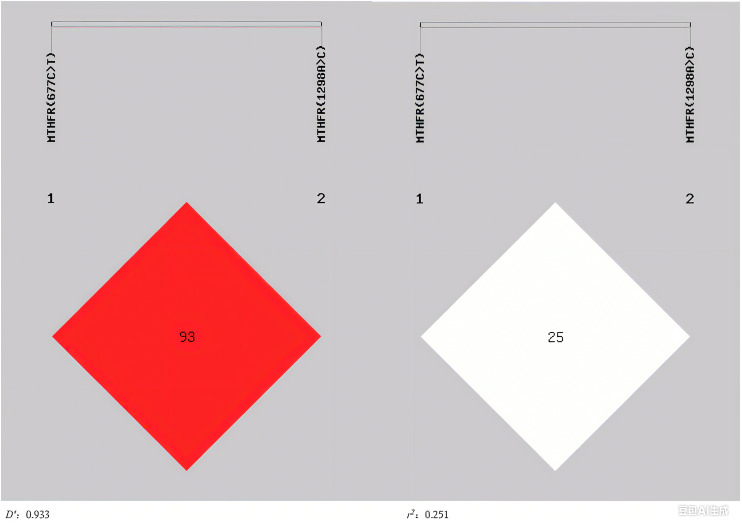
The linkage disequilibrium profiles of MTHFR (677C>T) and MTHFR (1298A>C).

Linkage disequilibrium (LD) between *MTHFR (677C>T*) and *MTHFR (1298A>C)* was analyzed using SHEsis online software (http://analysis.bio-x.cn/), where *D′* and *r*^*2*^ represent the degree of linkage disequilibrium (Ardlie, Kruglyak & Seielstad, 2002). The results showed that the 2 loci were in linkage disequilibrium (*D′* = 0.933) and the degree of correlation (*r*^*2*^ = 0.251) (Fig. 1). For haplotypes with a frequency >3%, there were three haplotypes involved in *MTHFR (677C>T)* and *MTHFR (1298A>C)*. The C-A haplotype was a protective factor for H-type hypertension (*P* = 0.028, *OR = 0.485*, 95%CI [0.252–0.934]), whereas the T-A haplotype was a risk factor (*P* = 0.022, *OR* = 2.029, 95%CI [1.096–3.756]) (Table 6).

**Table 6:** The association of MTHFR gene haplotypes with H-type hypertension.

Haplotype	Frequency		*OR* (95%CI)	*P*
	H-type hypertension group (*n* = 192)	Non-H-type hypertension group (*n* = 23)		
C-A	0.204	0.348	0.485 [0.252–0.934]	0.028
C-C	0.124	0.152	0.794 [0.336–1.876]	0.598
T-A	0.666	0.5	2.029 [1.096–3.756]	0.022

**Note:**

The haplotype order was *MTHFR (677C>T)* and *MTHFR (1298A>C)*.

### Distribution of *MTHFR* enzyme activity in the two groups

Based on the linked genotypes of *MTHFR (677C>T)* and *MTHFR (1298A>C)*, *MTHFR* enzyme activity can classify into four levels: normal enzyme activity (CC/AA), mild metabolic impairment (CC/AC), moderate metabolic impairment (CC/CC, CT/AA) and severe metabolic impairment (CT/AC, CT/CC, TT/AA, TT/AC, TT/CC) (Cai et al., 2024; Deng et al., 2015). There was no significant difference in the distribution of MTHFR enzyme activity between the two groups (*P* > 0.05) (Table 7).

**Table 7:** The distribution of MTHFR enzyme activity in the two groups.

Enzyme activity	*MTHFR (677C>T)/(1298A>C)*	Cases number *n* (%)	H-type hypertension group (%)	Non-H-type hypertension group (%)	*χ* ^*2*^	*P*
NEA		12 (5.6%)	10 (5.2%)	2 (8.7%)	4.321	0.229
	CC/AA	12 (5.6%)	10 (5.2%)	2 (8.7%)		
MIMI		11 (5.1%)	9 (4.7%)	2 (8.7%)		
	CC/AC	11 (5.1%)	9 (4.7%)	2 (8.7%)		
MOMI		65 (30.2%)	55 (28.6%)	10 (43.5%)		
	CC/CC	6 (2.8%)	6 (3.1%)	0 (0%)		
	CT/AA	59 (27.4%)	49 (25.5%)	10 (43.5%)		
EVMI		127 (59.1%)	118 (61.5%)	9 (39.1%)		
	CT/AC	32 (14.9%)	27 (14.1%)	5 (21.7%)		
	CT/CC	0 (0%)	0 (0%)	0 (0%)		
	TT/AA	93 (43.3%)	89 (46.4%)	4 (17.4%)		
	TT/AC	2 (0.9%)	2 (1.0%)	0 (0%)		
	TT/CC	0 (0%)	0 (0%)	0 (0%)		

**Note:**

NOEA, normal enzyme activity; MIMI, mild metabolic impairment; MOMI, moderate metabolic impairment; EVMI, evere metabolic impairment.

## Discussion

This study investigated the association between the genotypes of *MTHFR (677C>T)* and/or *MTHFR (1298A>C)* and H-type hypertension. Our research results indicate that the *MTHFR (677C>T)* TT genotype is an independent risk factor for H-type hypertension (*P* = 0.021, *OR* = 2.615, 95%CI [1.154–5.926]). Additionally, the C-A haplotype was a protective factor for H-type hypertension (*P* = 0.028, *OR* = 0.485, 95%CI [0.252–0.934]), while the T-A haplotype was a risk factor (*P* = 0.022, *OR* = 2.029, 95%CI [1.096–3.756]).

In the study, H-type hypertension patients accounted for about 89.3% of the hypertensive population, which is higher than the results of previous studies, which showed that H-type hypertension accounted for 75% of the hypertensive population in China (Chen et al., 2023). This discrepancy may be attributed to our relatively small sample size and regional population characteristics. In our study, the frequencies of the *MTHFR (677C>T*) wild-type homozygous CC, mutant heterozygous CT, and mutant homozygous TT genotypes are 13.5%, 42.3%, and 42.2%, respectively, with the T allele frequency of 65.3%, which was consistent with previous research in Shandong Province, where the T allele frequency was 63.1% (Fan et al., 2016). The frequencies of the *MTHFR (1298A>C)* wild-type homozygous AA, mutant heterozygous AC, and mutant homozygous CC genotypes are 76.3%, 20.9%, and 2.8%, respectively, with the C allele frequency of 13.3%. This was consistent with previous research results in Shandong Province, where the frequency of the C allele is 13.1% (Fan et al., 2016).

Previous studies have demonstrated that *MTHFR (677C>T)* polymorphism is associated with H-type hypertension in elderly individuals (*P* = 0.024, *OR* = 7.335, 95%CI [1.303–41.302]) (Zhang, Dou & Zhou, 2024). Additionally, the *MTHFR (677C>T)* allele mutation can increase the risk of ischemic stroke in older adults (Chang et al., 2019). In the codominant and recessive models, the *MTHFR (677C>T) TT* genotype was the strongest determinant of H-type hypertension (Ma et al., 2022). Our research results indicate that the *MTHFR (677C>T)* TT genotype was an independent risk factor for H-type hypertension (*P* = 0.021, *OR* = 2.615, 95%CI [1.154–5.926]), which was consistent with the results of previous studies. Currently, the results of studies on the association between the *MTHFR (1298A>C)* gene polymorphism and hypertension are still inconsistent (Liu et al., 2019; Mabhida et al., 2022; Yuan et al., 2019; Zhang et al., 2023b). These differences are likely due to ethnic and regional variations in study populations. Consistent with some of these reports, our study found no significant association between the *MTHFR (1298A>C)* polymorphism and H-type hypertension.

The results of linkage and haplotype analysis of *MTHFR (677C>T)* and *MTHFR (1298A>C)* show that there were seven kinds of haplotype combinations constructed by the 2 loci in the H-type hypertension group. CT/CC and TT/CC were not detected, with TT/AA being the most common (46.4%). In the non-H-type hypertension group, there were five haplotype combinations constructed by the 2 loci, CC/CC, CT/CC, TT/AC and TT/CC were absent, and the highest frequency was CT/AA (43.5%). These distributions are broadly consistent with prior findings (Fan et al., 2016). The results of our study showed that the *MTHFR (677C>T)* and *MTHFR (1298A>C)* were in linkage disequilibrium (*D′* = 0.933, *r*^*2*^= 0.251), aligning with earlier observations of LD between these loci (Ginani et al., 2023). For haplotypes with a frequency >3%, there were three haplotypes in *MTHFR (677C>T)* and *MTHFR (1298A>C)*. The C-A haplotype was a protective factor for H-type hypertension (*P* = 0.028, *OR* = 0.485, 95%CI [0.252–0.934]), while the T-A haplotype was a risk factor (*P* = 0.022, *OR* = 2.029, 95%CI [1.096–3.756]).

This study has certain limitations: Firstly, the sample size was small, and no genotypes with low mutation rates were not detected. Secondly, this study was a retrospective analysis. Thirdly, this study did not include healthy individuals for comparison. Future research will expand the sample size, conduct prospective cohort studies, and include healthy individuals as controls to enhance the reliability and persuasiveness of the results, thereby providing a scientific basis for personalized diagnosis and treatment.

## Conclusion

In patients with ischemic stroke, the *MTHFR (677C>T)* TT genotype was an independent risk factor for H-type hypertension. For haplotypes with a frequency >3%, there were three haplotypes in *MTHFR (677C>T)* and *MTHFR (1298A>C)*. The C-A haplotype was a protective factor for H-type hypertension, while the T-A haplotype was a risk factor.

In the future, we will expand the sample size, adopt a prospective cohort design, and include healthy individuals as controls to clarify the specific association between *MTHFR (677C>T)*, *MTHFR (1298A>C)* polymorphisms and related indicators and ischemic stroke. We will also explore individualized interventions for different genotypes, evaluate their effects, and provide scientific evidence for risk stratification and precision prevention and treatment of the population.

## Supplemental Information

10.7717/peerj.20210/supp-1 Supplemental Information 1 Dataset.

## Additional Information and Declarations

### Data Availability

The following information was supplied regarding data availability:

The raw data is available in the Supplemental File.
